# IEEE 802.15.4 Frame Aggregation Enhancement to Provide High Performance in Life-Critical Patient Monitoring Systems

**DOI:** 10.3390/s17020241

**Published:** 2017-01-28

**Authors:** Muhammad Sajjad Akbar, Hongnian Yu, Shuang Cang

**Affiliations:** 1 Faculty of Science and Technology, Bournemouth University, Poole BH12 5BB, UK; makbar@bournemouth.ac.uk; 2 Faculty of Management, Bournemouth University, Poole BH12 5BB, UK; scang@bournemouth.ac.uk

**Keywords:** patient monitoring systems, WBASN, QoS, IEEE 802.15.4, IEEE 802.15.6, frame aggregation, ECG, duty cycle, energy efficiency

## Abstract

In wireless body area sensor networks (WBASNs), Quality of Service (QoS) provision for patient monitoring systems in terms of time-critical deadlines, high throughput and energy efficiency is a challenging task. The periodic data from these systems generates a large number of small packets in a short time period which needs an efficient channel access mechanism. The IEEE 802.15.4 standard is recommended for low power devices and widely used for many wireless sensor networks applications. It provides a hybrid channel access mechanism at the Media Access Control (MAC) layer which plays a key role in overall successful transmission in WBASNs. There are many WBASN’s MAC protocols that use this hybrid channel access mechanism in variety of sensor applications. However, these protocols are less efficient for patient monitoring systems where life critical data requires limited delay, high throughput and energy efficient communication simultaneously. To address these issues, this paper proposes a frame aggregation scheme by using the aggregated-MAC protocol data unit (A-MPDU) which works with the IEEE 802.15.4 MAC layer. To implement the scheme accurately, we develop a traffic patterns analysis mechanism to understand the requirements of the sensor nodes in patient monitoring systems, then model the channel access to find the performance gap on the basis of obtained requirements, finally propose the design based on the needs of patient monitoring systems. The mechanism is initially verified using numerical modelling and then simulation is conducted using NS2.29, Castalia 3.2 and OMNeT++. The proposed scheme provides the optimal performance considering the required QoS.

## 1. Introduction

Patient monitoring using biomedical sensors is a popular body area sensor network (WBASN) application that can to continuously monitor chronic and non-chronic diseases. WBASNs usually work in a star topology under the central control of a coordinator. A patient monitoring system is comprised of different sensors which measure physiological parameters including electrocardiography (ECG), electroencephalography (EEG), electromyography (EMG), accelerometry, gyroscopy, pulse oximeters, blood pressure, temperature, barometry and heart rate monitoring, etc. The sensors periodically collect data from the body and send it to a monitoring station through a coordinator node [1]. The periodic data from the sensors have different characteristics in the context of delivery delay and sensing rate. There are two basic reasons for the different characteristics of periodic data collected from various sensors; the first is collection from diverse physiological sources like blood pressure, ECG and temperature, etc., the second is the default behavior of the sensor.

The efficient performance of WBASNs for patient monitoring systems requires a combined set of QoS including time bounded delays, reliability, appropriate throughput and energy efficiency. Besides the continuous monitoring of the periodic data from sensors, there is also exist some high priority data traffic that requires guaranteed services like ECG data, etc. Similarly, event-based emergency data may be generated at any time, e.g., when a sensor detects a stroke episode. The periodic data, high prioritized data and emergency data demand different sets of QoS [2,3].

Therefore, the design of MAC protocols plays a vital role to provide appropriate communication services to the diverse sensors used in patient monitoring systems. Time-division multiple access (TDMA) and carrier sense multiple access with collision avoidance (CSMA/CA) are two basic MAC mechanisms used in WBASNs to support periodic and urgent traffic [4]. Usually, the CSMA/CA is considered to be appropriate for low, urgent, adaptive and scalable traffic patterns, whereas the TDMA is recommended for periodic traffic [5,6,7]. In this context, various hybrid MAC protocols are proposed [8,9,10,11,12,13,14,15,16,17,18,19,20,21,22,23,24,25,26,27,28,29] based on IEEE 802.15.4 and IEEE 802.15.6 standards. These protocols can provide both channel access options i.e., CSMA/CA and TDMA. Usually, these protocols use time synchronization mechanisms to incorporate energy efficient mechanisms. The IEEE 802.15.4 defines a superframe structure containing a contention-free period (CFP) and a contention-access period (CAP). In the CAP, nodes deliver content for the channel using the slotted CSMA/CA, whereas CFP mode works in TDMA manner where the coordinator can assign maximum seven guaranteed time slots (GTS) to a request node. The WBASNs for patient monitoring systems deal with diverse bio-medical sensors which require services for periodic or urgent data. However, issues still exist with these hybrid protocols in terms of provision of a combined set of QoS including limited delay, reliability, appropriate throughput and efficient energy utilization for patient monitoring systems. The issues include random selection of backoff exponent BE without considering network conditions, MAC unreliability problems, lack of dynamic adaptive capabilities, unfair under saturation conditions, superframe duration selection, traffic priorities issues and the limited number of GTS slots etc. Table 1 provides the requirements of communication analysis for different bio-medical sensor devices. 

In this paper, a different approach is used to address the above issues and to optimize the performance of existing hybrid MAC protocols for patient monitoring systems. A frame aggregation mechanism is proposed at the MAC layer which will send multiple MAC frames under the single PHY header within a single successful channel access. This paper implements this idea for IEEE 802.15.4. The aggregation mechanism will reduce the overheads, i.e., the waiting time before a successful channel access and underutilization of the channel capacity. A successful channel access transmits multiple frames which mean we not only send multiple frames in cost of a single frame overhead but also with a lesser PHY header overhead. The aggregation mechanism significantly reduces the delay and increases the throughput for patient monitoring systems where many small packets are generated. Moreover, the overall network lifetime is increased due to overhead avoidance of aggregated packets. This research initially defines the traffic patterns for such biomedical sensor networks for patient monitoring systems. These traffic patterns provide the requirements on how much and how frequent data is generated. These requirements elaborate about the channel access necessities for such applications. Then based on obtained information a channel modeling is conducted to define the problem statement of the research work. The proposed frame aggregation solution uses the information achieved from the traffic patterns to clearly define the aggregation limits. In the later section, a detailed discussion is provided on how the proposed frame aggregation mechanism can be incorporated with the superframe structure of IEEE 802.15.4. The evaluation is conducted based on mathematical modeling and simulations.

## 2. Related Work

MAC protocols have a vital role for the efficient functionality of WBASNs. The efficient MAC layer protocols for WBASNs have been a hot research topic for past few years. Aiming at provision of specified QoS in terms of delay, throughput, reliability and energy many efforts have been made to develop a new improved MAC protocol based on existing standards i.e., IEEE 802.15.4 and IEEE 802.15.6. Based on the literature, we summarize a comparative analysis of the protocols in term of their QoS support in Table 2, where D represents delay, R reliability, E energy efficiency, T throughput, C collision handling and S scalability. IEEE 802.15.6 is known as the WBAN standard and theoretically claims to provide a rich communication suite that is suitable for different kind of WBAN applications [31]. However, it only elaborates the operational details to interoperate the WBAN devices and leave the implementation on users per their application needs. In this context, the MAC scheme does not reflect a complete MAC protocol [32]. There are some other hybrid approaches which incorporate the contention mechanism over reservation mechanisms to improve the performance of critical-applications [33].

Table 2 lists the literature of the related protocols with the information regarding QoS support. To improve the IEEE 802.15.4, an enhanced version of IEEE 802.15.4 in 2012 and named IEEE 802.15.4e were published. This enhancement mainly introduces three behavioral modes to improve the channel access mechanisms of the existing IEEE 802.15.4 standard [37,38]. These modes include time slotted channel hopping (TSCH), deterministic and synchronous multi-channel extension (DSME) and low-latency deterministic networks (LLDN). TSCH is considered as a new efficient MAC protocol that incorporates time-slotted access with multiple channel and channel hopping. It is popular mainly due to its interoperability with the Internet of Things (IoTs) with appropriate support to mesh networks. As TSCH more suitable for high coverage scenarios particularly node-coordinator scenarios like patient monitoring systems are ignored. The DSME is proposed for applications which require time bounded services with high reliability, especially for industrial and healthcare applications. It is the improved form of IEEE 802.15.4 which provides the adoptability option for time-varying traffic which is lacking in the original standard. DSME improves the performance of 802.15.4 by increasing the number of GTS slots and operating frequency channels. Moreover, it introduces the concept of the multi-superframe structure which is capable of handling both periodic and aperiodic traffic. The best channel is selected to provide the high reliability. In multi-superframe, various CFP and CAP periods are aggregated. However, DSME lacks implementation examples due to its complex mechanisms. Further the nodes are required to stay in the active mode which increases the energy utilization. LLDN is a TDMA based mechanism and depends on a simple superframe structure where each node can get the exclusive access for a timeslot [39]. However, LLDN is not suitable for time-varying traffic; moreover, it requires information regarding number of nodes and packet size. In [35], a decentralized approach DT-SCS an improved version of TSCH is presented by using the pulsed-couple oscillators that simultaneously do the synchronization and desynchronization over multiple channels. This approach is more suitable for vehicular environment, whereas in WBASNs a centralized approach with a controller/coordinator is more successful; moreover, channel switching mechanism is still a source of delay which is a challenge for medical applications. The reinforcement learning based approach [36] is presented for a distributed environment; this approach provides energy efficient communication while maintain the high throughput. However, the computational complexity is involved which is less likely for WBASNs in terms of short memory of sensors and the requirement of limited delay. In [40] the authors describe the basic requirements of a body sensor network (BSN)-based software framework for signal processing applications. Signal processing in a node environment (SPINE) is proposed as an efficient framework for signal processing applications for BSN-based networks. SPINE is tested on hardware and software platforms. In [41] a detailed survey of BSN is provided in the context of fusion of the data from multiple heterogeneous biomedical sensor devices located around the body. The paper specifically discusses the issues of physical activity recognition; moreover, it provides the comparison of different existing platforms based on distinctive properties that affect the design of data fusion.

Due to the abovementioned limitations and complex solutions, this paper proposes using the frame aggregation mechanism with existing MAC of IEEE 802.15.4 to optimize the performance of the standard in terms of time bounded services, appropriate throughput, reliability, and energy efficiency. 

## 3. Proposed Design

We start with the identification of traffic patterns for patient monitoring systems in WBASNs. For this research, we use nine types of bio-medical sensor nodes as mentioned in Table 1. Based on traffic patterns requirements, we map them on the MAC layer to figure out the performance gap. We then propose the frame aggregation mechanism and incorporate it with the IEEE 802.15.4 superframe structure. We consider a star topology network with sensor nodes. The description of each sensor with its traffic pattern is presented below.

### 3.1. Application Traffic Patterns Identification

In this section, traffic patterns of different bio-medical sensor devices will be computed [42]. 

#### 3.1.1. Electrocardiography (ECG)

An ECG is a waveform that shows the transmission of electric potentials via heart muscles in the context of time. Overall, an ECG waveform represents a non-invasive way of the viewing heart function. A standard ECG output is obtained through 12 leads, however a wireless sensor uses less number of leads. From Table 1 [30], considering a sampling rate of 250 Hz and sampling resolution of 16 bits, we have sample interval time (SIT) = 1/250 = 4 ms and maximum possible data size (MPDS) = sampling rate × resolution = 250 × 16 = 4 Kbps = 500 bytes/s. Thus, the data need to send (DNS) in 4 ms can be computed as:

(1) $$\mathrm{DNS}\;=\frac{\mathrm{MPDS}\times 4}{1000}=2\;\mathrm{bytes}$$

In this paper, we consider the minimum MAC 9 bytes overhead. Equation (2) shows a complete packet size which is sent in the ECG node case:

(2) DNS after 4 ms including minimum MAC and physical (PHY)header=2 (data)+9 (MAC header)+6 (physical layer header)=17 bytes=136 bits

Equation (3) computes the required data rate:

(3) Total data rate required by ECG packet=Sampling rate (250)×packet size (136)=34 Kbps

Equations (1)–(3) show that after each 4 ms, a specific amount of the data will be ready for transmission. In the slotted CSMA/CA case, whenever this gets the channel and only transmits the single packet if the remaining CAP is enough to transmit that packet. 

#### 3.1.2. Electromyography (EMG)

EMG helps to understand the muscle function with the help of electrical signals generated by the muscles. Surface electrodes are placed on the skin and these electrodes can receive electrical signals when a muscle performs some movement like contracting. Hence, EMG is helpful for measuring muscle activity and used in specific bio-medical activities. 

From Table 1, if we consider sampling rate 150 Hz and the sampling resolution 12 bits then SIT = 1/150 = 6 ms; MPDS = 150 × 12 = 1.8 Kbps = 225 bytes/s; using Equation (1) the DNS after 6 ms = (225 × 6)/1000 = 1.3 bytes; using Equation (2) in case of EMG node, DNS after 6 ms including minimum header size = 1.3 + 9 + 6 = 16.3 bytes = 131 bits; using Equation (3), total data rate required by EMG packet = 150 × 131 = 19.6 Kbps.

#### 3.1.3. Electroencephalography (EEG)

EEG signals represent the brain’s electrical activity. Currently, ambulatory EEG (AEEG) recordings are valuable in the diagnosis of epilepsy and monitoring patient response to therapy. To obtain the EEG information in the normal routine wireless ECG sensor is very useful. 

Sampling rate is 250 Hz and sampling resolution is 16 bits; using Equations (1)–(3), following values are computed from the EEG data: SIT = 1/250 = 4 ms; MPDS = 250 × 16 = 4 Kbps = 500 bytes/s; DDNS after 4 ms = (500 × 4)/1000 = 2 bytes; DNS after 4 ms including minimum header = 2 + 9 + 6 = 17 bytes = 136 bits; Total data rate required by ECG packet = 250 × 136 = 34 Kbps.

#### 3.1.4. Pulse Oximeter

A pulse oximeter is helpful for measuring the heart rate, oxygen saturation levels (SpO2) and blood volume that is related with the cardiac cycle. It can be placed on an earlobe or finger and it is composed of the infra-red light-emitting diodes (LEDs) and a photodetector which is useful to measure oxygenated hemoglobin to deoxygenated hemoglobin. From Table 1, considering sampling rate 100 Hz and the sampling resolution 12 bits, we have SIT = 10 ms; MPDS = 150 bytes/s; DNS after 10 ms including minimum header size = 132 bits; Total data rate required by Pulse oximeter packet = 13.2 Kbps. 

#### 3.1.5. Blood Pressure (BP)

BP is the measurement of the pressure of circulating blood on the walls of vessels. During a cardiac cycle, BP fluctuates between a maximum (systolic) and a minimum (diastolic) pressure. BP readings have a significant relation with cardiovascular events; therefore, BP is an important parameter for monitoring activity of patients. Considering sampling rate 100 Hz and the sampling resolution 12 bits we have SIT = 10 ms; MPDS = 150 bytes/s; DNS after 10 ms including minimum header size = 16.5 bytes = 132 bits; Total data rate required by Pulse oximeter packet = 13.2 Kbps.

#### 3.1.6. Respiration

A patient’s respiratory status is helpful to measure a few important health indicators like short of breath, changes in skin color, general pallor and partial or complete loss of consciousness. Therefore, respiratory status monitoring of a patient is an essential aspect in the healthcare domain. From Table 1, considering sampling rate 25 Hz and the sampling resolution 8 bits we have SIT = 40 ms; MPDS = 25 bytes/s; DNS after 40 ms = 1 byte; DNS after 40 ms including minimum header size = 128 bits; Total data rate required by respiration packet = 3.2 Kbps.

#### 3.1.7. Blood Glucose

The blood glucose level determines the amount of glucose (sugar) present in the blood. It is observed that the risk for hyperglycemic excursions for individuals with type 1 diabetes can be reduced by continuously monitoring the glucose level. Moreover, the required dose of insulin can be estimated. Using implantable wireless sensor, the glucose level can be monitor. From Table 1, considering sampling rate 0.004 Hz and sampling rate 8 bits we have SIT = 250,000 ms; MPDS = 0.004 bytes/s; DNS after 250,000 ms = 1 byte; DNS after 250,000 ms including minimum header size = 132 bits; Total data rate required by Pulse oximeter packet = 528 bps. 

#### 3.1.8. Skin Temperature

Human body temperature, also called normothermia, is composed of a defined temperature band and depends upon factors like health, age, place, time and emotional status etc., of a person. Its range is well defined and 37.0 °C (98.6 °F) is considered as normal temperature. It is widely used in patient monitoring systems. Considering the sampling rate is less than one in 60 s we have SIT = 60,000 ms; MPDS = 0.033 bytes/s; DNS after 60,000 ms = 1.98 bytes; DNS after 60,000 ms including minimum header size = 136 bits; Total data rate required by accelerometer packet = 2.27 Kbps. 

#### 3.1.9. Accelerometer

An accelerometer is used to measure the acceleration of an object. In patient monitoring systems, it is attached with the patient to get location information in case of mobility. Considering sampling rate 25 Hz and the sampling resolution 12 bits we have SIT = 40 ms; MPDS = 37.5 bytes/s; DNS after 40 ms = 1.5 byte; DNS after 40 ms including minimum header size = 132 bits; Total data rate required by the accelerometer packet = 3.3 Kbps. Table 3 presents the summary of traffic patterns for a patient monitoring system.

### 3.2. Problem Formulation Using Traffic Patterns

**Figure 1:**
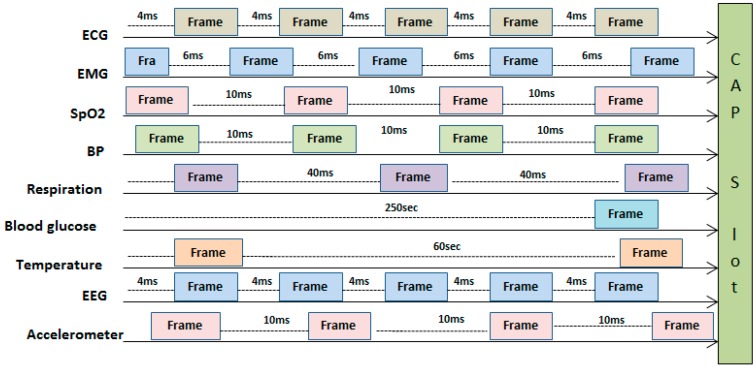
Channel access mapping of bio-medical devices for slotted CSMA/CA in a CAP slot.

We select IEEE 802.15.4 for the experiments and implementation due to its simple hybrid channel access mechanisms. IEEE 802.15.4 operates under a superframe which can be divided in active and inactive modes. The active mode consists of two basic periods, including CAP and CFP. CAP consists of time slots which can be acquired through slotted CSMA/CA, whereas CFP provides GTS slots and works in TDMA manner. Both periods work in an energy efficient manner by using synchronization mechanisms [1,2]. CAP follows several steps in the contention process. In the first step, the slotted algorithm for CSMA/CA initializes the values of the number of backoff attempts (NB), contention windows (CW) and backoff exponent (BE). The node waits according to its selected backoff period and then performs clear channel access (CCA). In the 1st set of CCA attempts, if the channel is found idle then CW is decremented from 2 to 1 and the node performs the 2nd CCA. If the channel is idle in the 2nd CCA and the value of CW decremented from 1 to 0 then CCA is assumed to be successful and the data is transmitted else the CCA process starts again. In the case that the channel is sensed busy in both CCA attempts, NB and BE are incremented by one, whereas the value of CW is initiated from 2. Figure 1 shows the scenario of channel access for a single CAP slot. The biomedical sensor devices generate frames according to their sampling interval shown in Table 3 and try to access the channel through contention mechanism of slotted CSMA/CA. When both modes i.e., CAP and CFP are used together then in the CFP mode GTS slots are assigned to important periodic data like ECG, EMG and EEG, whereas other nodes which require event bases or emergency data use the CAP mode.

**Figure 2:**
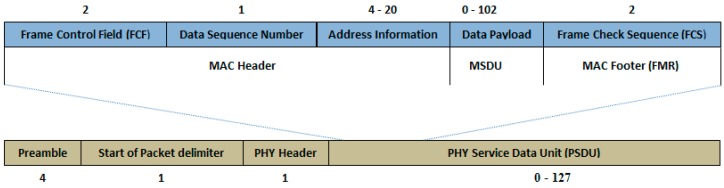
Frame structure of IEEE 802.15.4 [2].

The IEEE 802.15.4 standard supports the maximum frame size up to 127 bytes including 25 bytes of MAC header and 102 bytes of payload. Figure 2 illustrates the frame structure of IEEE 802.15.4.

**Figure 3:**
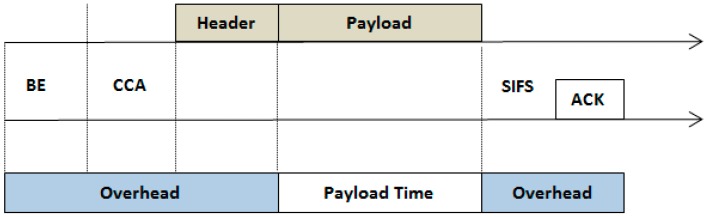
Overheads in successful packet transmission [3].

A successful packet transmission of IEEE 802.15.4 with its overhead is illustrated in Figure 3. By analyzing the single successful packet transmission mechanism, overhead for a successful channel access at different stages can be observed. It includes random backoff delay, two times CCA, 802.15.4 header with each frame, SIFS and acknowledgement transmission time [1].

**Figure 4:**
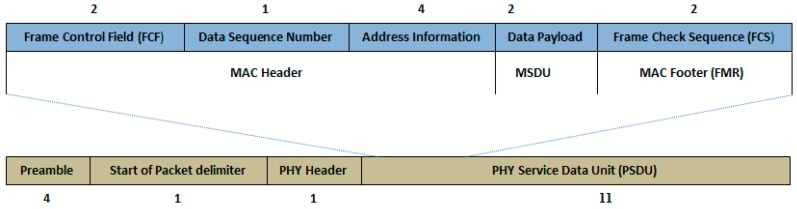
ECG frame structure.

The maximum packet size from these bio-medical sensor devices used in the patient monitoring system is 17 bytes for a single frame including headers as shown for ECG in Figure 4. The size is almost the same for other used devices; therefore, we consider the total packet size for these devices as 17 bytes in our experiments. Each frame will content for the channel to send the data, so a node which wins access to the channel transmits a small packet and for the next packet transmission, it needs to contest for the channel again. The common characteristic of these biomedical sensor devices is that they generate many small packets in a limited time. Moreover, the generated data from these devices require a combined set of QoS including limited delay, specified throughput, reasonable reliability and energy efficiency. By analyzing the packet transmission process of IEEE 802.15.4, it is noted that CFP and CAP provide inefficient channel utilization for the patient monitoring biomedical sensor devices, especially when the duty cycle mechanism is used [31]. Thus, using equations in Section 3.1 we have DNS after 4 ms including header = 2 (data) + 9 (minimum MAC layer header) + 6 (physical layer header) = 17 bytes.

Further, in patient monitoring systems the traffic pattern of sensor nodes is periodic, which means these nodes generate many packets continuously in a limited time. Due to such a traffic pattern, usually a node has multiple packets in queue for transmission; whereas if it gets a successful channel access it can transmit one packet. Therefore, on getting a channel access, a transmission node is under utilizing the available maximum packet size capacity which is 127 bytes for a MAC frame and only transmits up to 17 bytes shown in Figure 1. When the payload size is small, it faces a larger overhead, especially due to pre-defined overheads of the channel access mechanism. 

### 3.3. Proposed Solution

Following the discussion in Section 3.2, it is observed that wireless biomedical sensors generate a large number of small packets in a limited time period which poses challenges for the MAC layer, especially on channel access process. The main issue is the involvement of overheads for a single tiny frame transmission. These overheads include the waiting time until a successful channel access and the underutilization of the channel after successful channel access. In the case of the IEEE 802.15.4 slotted mode, the waiting time consists of the random backoff exponent generated time, CCA, CW, acknowledgement waiting time, SIFS time and turnaround time. The underutilization of the channel refers that a single frame in the case of patient monitoring applications consists of 2 bytes data with minimum 9 bytes MAC header and 6 bytes PHY header and transmits 17 bytes on a channel which provides the maximum capacity of 133 bytes including MAC and PHY headers. 

To address these issues, we propose to use a frame aggregation mechanism, which sends multiple MAC frames under a single PHY header within a single successful channel access. The concept of frame aggregation is successfully used in IEEE 802.11n [43,44] and makes a huge difference in throughput and delay performance by efficiently utilization in channel access mechanism.

**Figure 5:**
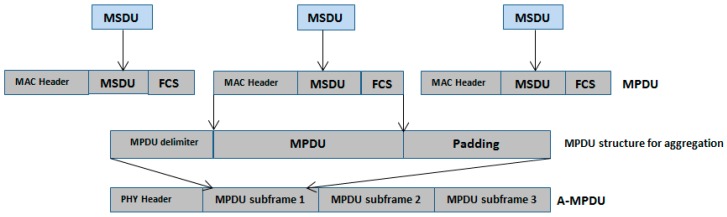
Frame aggregation mechanism.

This helps to reduce the overheads, i.e., the waiting time before a successful channel access and underutilization of the channel capacity. Figure 5 presents the frame aggregation mechanism in detail. As in a single channel, access multiple frames are transmitted which means we send multiple frames in the cost of a single frame overhead with lesser PHY header overhead. Moreover, aggregating the frame under a single PHY header almost fully utilizes the channel. In the following section, we present the proposed design and its evaluation and validation. 

**Figure 6:**
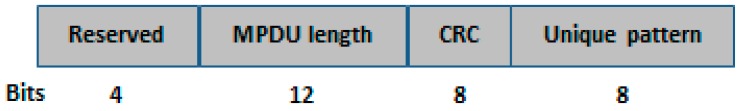
Delimiter structure.

Figure 5 presents the design of the frame aggregation mechanism at the MAC layer. Initially, the data is received in the form of MSDUs. After applying the MAC headers, it becomes MPDU and ready for the physical layer. The combination of multiple MPDUs creates aggregated MPDU (A-MPDU). A-MPDU is generated before passing to the PHY layer for final transmission. The MAC does not wait for a certain number of MPDUs to create A-MPDU, so if a node gets a channel access the MAC takes available MPDUs to make A-MPDU for transmission. The destination of the all MPDUs must be same. The maximum size for an A-MPDU frame must not exceed than 127 bytes. Each encapsulated MPDU consists of a delimiter at the start and padding bits at the end. The purpose of the delimiter is to define a separation pattern of individual MPDU in A-MPDU. The delimiter represents the information including MPDU length, reserved bits (first four bits for future use), cyclic redundancy checks (CRC) and unique pattern. MPDU Length field represents the length of the MPDU. CRC design uses reserved and MPDU length fields for its calculation. The unique pattern is helpful to find the next delimiter. Figure 6 presents the delimiter structure.

In this aggregation mechanism, selective retransmission is possible due to the presence of an individual frame check sequence (FCS) for each MPDU. All the MPDUs in an A-MPDU have the same traffic identifier (TID) to effectively work with block acknowledgement (BA) mechanism. In the de-aggregation process, the receiving node first validates the CRC integrity, in the case of successful CRC check, the A-MPDU is de-aggregated and data is passed to the application layer. 

**Figure 7:**
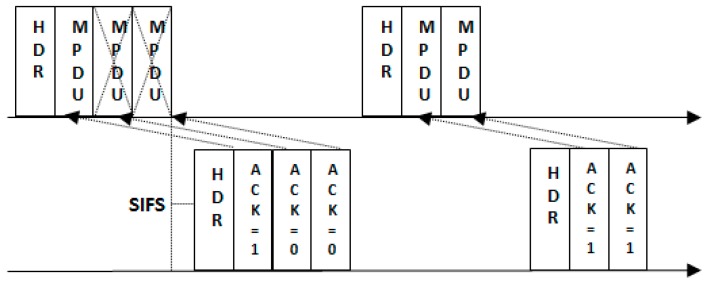
Block Acknowledgement procedure.

The block acknowledgement mechanism is widely used with IEEE 802.11n to support the frame aggregation mechanism. The IEEE 802.15.4 already gives the option to use a block acknowledgement [37]. The A-MPDU are capable of enhancing channel access performance significantly; however, in the wireless environment, the chances of frame error get high by increasing the frame size. Moreover, if the bit-error-rate (BER) is high then the probability of packet loss rate is also high which will cause high retransmissions. To resolve this issue, the block acknowledgement mechanism is used to support the A-MPDU frame. The receiving node receives the A-MPDU and only sends a collective acknowledgement against those MPDUs which are received correctly. The sending node will only retransmit the specific MPDU. The use of block acknowledgement mechanism supports the frame aggregation mechanism in high BER environment. Figure 7 shows an example of a block acknowledgement mechanism.

### 3.4. Numerical Analysis

In the following, the analysis regarding frame aggregation effect on the performance of IEEE 802.15.4 by incorporating traffic patterns of patient monitoring systems is conducted. The required frame size of MPDU with minimum header overhead is:

(4) MPDU Size=2 (data)+9(Minimum MAC layer header)=11 bytes

The size of single aggregated MPDU (A-MPDU) frame size is:

(5) A−MPDU frame size=4 bytes (delimiter size)+11 bytes (MPDU)+4 bytes (padding)=19 byte

The total A-MPDU packet size is:

(6) A− MPDU packet size=19 bytes+6 bytes (PHY header)=25 bytes

Similarly, size of A-MPDU with two frames is

(7) A−MPDU =19 bytes+19 bytes+6 bytes (PHY header)=44 bytes

Considering the maximum packet size capacity 127 bytes and the size of single A-MPDU from Equations (5) and (6), we aggregate the maximum six MPDU in an A-MPDU. Therefore, the size of the maximum A-MPDU is:

(8) Total size of A− MPDU =(6×19)+6 bytes (PHY header)=120 bytes

Transmission time of a maximum-sized A-MPDU [31] is:

(9) $$T_{\mathrm{frame}}\;\mathrm{for}\;A-\mathrm{MPDU}=\frac{\left(120\right)\times 8}{\mathrm{data}\;\mathrm{rate}}=3.84\;\mathrm{ms}$$

In the following, we compute the value of total delay using [1,31]:

(10) $$\mathrm{ED}=\mathrm{macMaxFrameRetries}\times \left(T_{\mathrm{WT}}+T_{\mathrm{frame}}+T_{\mathrm{Ack}}+T_{\mathrm{SIFS}}+{\;T}_{\tau A}\right)$$

For the scenario mentioned in Figure 1 where multiple nodes try to access the channel, we compute the value of delay by considering the default MAC attribute values mentioned in Table 4. 

This includes total waiting time ($T_{\mathrm{WT}}$), where $T_{\mathrm{WT}}$ represents the backoff time period which is assigned to a node before going to channel access attempt. Equation (10) also includes frame transmission time ($T_{\mathrm{frame}}$), acknowledgement receiving time ($T_{\mathrm{Ack}}$), short interframe space time ($T_{\mathrm{SIFS}}$) and turnaround time ($T_{\tau A}$). For analysis, we consider the bit rate of 250 kbps (2.4 G Hz frequency band is considered with BPSK modulation and a symbol represents 4 bit).$\;T_{\mathrm{BOslot}}$ denotes the time for a single backoffslot:

(11) $$T_{\mathrm{WT}}=\mathrm{macMaxCSMABackoffs}\times \left(T_{\mathrm{BE}}+T_{\mathrm{CCA}\;}\right)+{\;T}_{\mathrm{CW}}$$

where $T_{\mathrm{BE}}$ is the random backoff time, $T_{\mathrm{CCA}\;}$the clear channel assessment time and $T_{\mathrm{CW}}$ the contention windows time. The random backoff time ($T_{\mathrm{BE}}$) is the waiting time for channel access which is computed as:

(12) $${\;T}_{\mathrm{BE}}=(2^{\mathrm{macMinBE}}-\;1)\times {\;T}_{\mathrm{symbol}}\;\left(\mathrm{aUnitbackofftime}\right)$$

**Figure 8:**
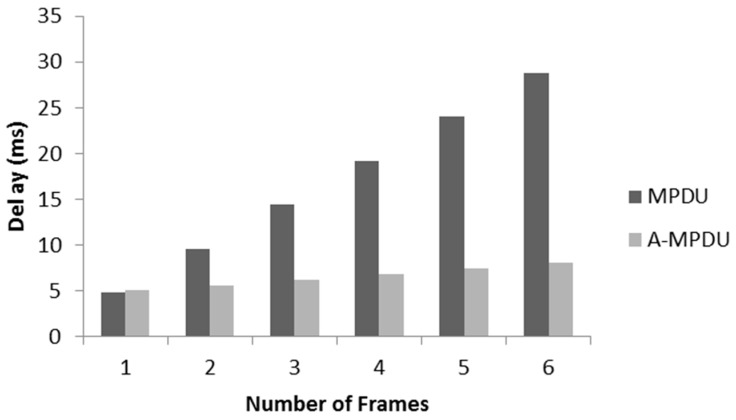
Delay comparison between MPDU and A-MPDU.

Using Equation (12) with ideal channel conditions, Figure 8 presents a comparative analysis of total delay for the transmission of the number of frames with aggregation and without aggregation. It is noted that without aggregation, delay continuously increases as every frame takes a separate channel access attempt and its overheads. We assume that a node gets channel access attempt at the transmission time. On the other hand, for the A-MPDU, a node sends multiple packets in single channel access attempt, only the packet size varies by increasing the frame aggregation size. It can be concluded that if there is a need to send 6 frames, an A-MPDU can sent it in one channel access with a minimum delay as in the cost of one frame it is sending five more frames. In contrast, the MPDU takes at least six channel attempts and the delay is significantly high. We also incorporate this analysis in our simulations.

**Figure 9:**
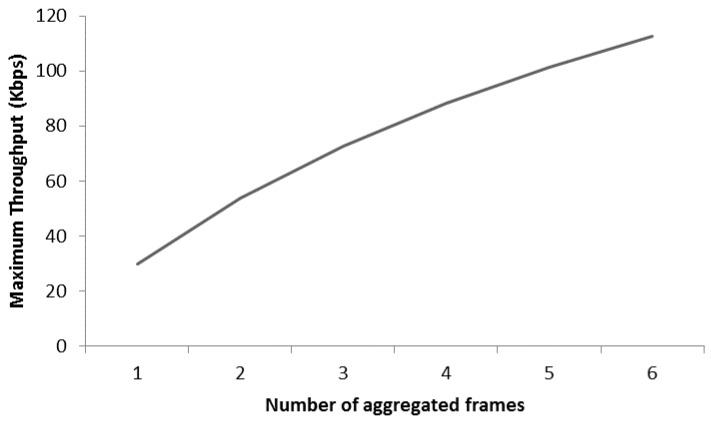
Maximum throughput analysis.

Figure 9 presents the MT analysis for the same scenario mentioned in Figure 8. It is shown that MT increases with the increase of aggregation level like from two frames in an A-MPDU to maximum of six frames. Therefore, it is concluded that, frame aggregation mechanism enhances the packet delivery which is helpful to reduce the delay and provision of time bounded services in WBASNs. The maximum throughput (MT) is considered as the ratio of payload size (bytes) to the transmission delay for specific payload size and is computed as:

(13) $$\mathrm{MT}=\frac{\left(x\right)\times 8}{\mathrm{transmission}\;\mathrm{delay}}$$

### 3.5. Mapping of Proposed A-MPDU Mechanism with Superframe

To adjust the aggregation mechanism with superframe of IEEE 802.15.4, we need to compute such the superframe duration (SD) where at least one or more than one A-MPDU could transmit. The possible maximum size in our scenario is 120 bytes as mentioned in Equation (8) and the transmission time for a single A-MPDU is 3.84 as mentioned in Equation (9). The superframe duration (SD) represents the duration of superframe which consists of 16 time slots [31] is:

(14) $$\mathrm{SD}\;=\;\mathrm{aBaseSuperframeDuration}\;\times \;2^{\mathrm{SO}}\;\mathrm{symbols}$$

where superframe order is represented by SO. aBaseSuperframeDuration is calculated as

(15) aBaseSuperframeDuration=aBaseSlotDuration × aNumSuperframeSlots × symbolTime

Symbol time using 2.4 GHz frequency band and symbol rate 4 is

(16) symbolTime = 1 / (phyDataRate × 1000 / phyBitsPerSymbol)symbolTime = 1 / ( 250× 1000 / 4) = 0.016 msThe default value of aBaseSuperframeDuration with (SO=0)= 60× 16× 0.016 =15.36 ms

The single slot time should be at least 3.84 ms as mentioned in Equation (9). Using Equation (14) the suitable value of SD is computed which fulfils the slot duration requirement.

$$\mathrm{SD}\;=\;15.36\times \;2^{2}=61.44\;\mathrm{ms}$$

The single slot duration is computed as

(17) Single slot duration = Total SD time/Number of slots = 61.44/16= 3.84 ms

It is noted that 3.84 ms is the same time as computed in Equation (9), which means that SO should be at least 2 or greater than 2 so that a complete A-MPDU could be transmitted. 

## 4. Performance Evaluation, Analysis and Discussion

The aim of proposing the use of frame aggregation mechanism for the patient monitoring system is to provide a combined set of QoS for the periodic data generated by the bio-medical sensor devices. Here, the QoS set refers towards provision of unbounded delay, appropriate throughput, reliability and energy efficiency. The delay mentioned in the graphs represents the average delay. Some of these bio-medical sensors like ECG and EEG etc., generate many small packets in a short time interval. The aggregation mechanism for IEEE 802.11n is implemented in NS-2.29 at the MAC layer. We conducted the capacity analysis of different aggregation in NS-2.29 and later we used the results while doing our evaluation in Castalia 3.2 and OMNeT++. A star topology is considered where it is assumed that there are 10 nodes initially. Node 0 is configured as the coordinator node. We used both available modes for the channel access including CFP and CAP. Table 5 provides the values for simulation parameters.

**Figure 10:**
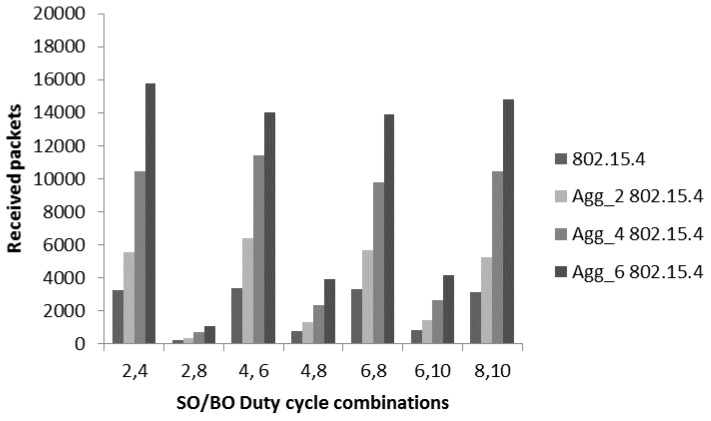
Received packets comparison.

Figure 10 provides the received packets comparative analysis of 802.15.4 with the aggregated-802.15.4. The focus of evaluation is to explore the capacity of different level of aggregations. In the simulation, for aggregated-802.15.4, we used different frame levels including Agg_2 802.15.4 (two frames in an A-MPDU), Agg_4 802.15.4 (four frames in an A-MPDU) and Agg_6 802.15.4 (six frames in an A-MPDU). The purpose of using different levels of aggregation is to conduct a detailed performance analysis of the aggregation mechanism. As duty cycle (DC) mechanism is an integral part of IEEE 802.15.4 to provide energy efficient data transmission, the duty cycle is [4,31]:

(18) $$\mathrm{DC}=\;\frac{2^{\mathrm{SO}}}{2^{\mathrm{BO}}}$$

The beacon order (BO) represents the total duration of superframe including active and inactive periods. Therefore, different combinations of the duty cycle are considered including 2/4 (25%), 2/8 (2%), 4/6 (25%), 4/8 (6.25%), 6/8 (25%), 6/10 (6.25%) and 8/10 (25%). The value 25% means that a node stays awake for 25% in the superframe duration. In all combinations, we set SO = 2 or more than 2 to achieve the appropriate superframe duration as derived in Section 3. Moreover, we also used a variety of SO values like 2, 4, 6 and 8 to see the optimal results as the superframe duration is an important aspect for throughput provision. In Figure 10, we considered slotted CSMA/CA mode of CAP period with different combination of duty cycle and evaluated the number of received packets with and without aggregation mechanisms. Initially, DC with values 25% shows the higher number of received packets is near 3000. It is obvious that data generation is high at the application layer, however due to the duty cycle mechanism used to save the energy a node stays awake for a limited time. The packets keeps coming to the queue and a limited number of packets are sent from the queue. This behavior creates the performance bottle-neck for patient monitoring systems, where many small packets are generated in a short time. To overcome this issue, we used the different levels of proposed aggregated mechanisms. Overall, aggregation mechanism is helpful to increase the number of received packets. For the 1st combination 2/4 (25%), the value of received packets for non-aggregated 802.15.4 is 3000 which increase up to 5566, 10,443 and 15,771 for Agg_2 802.15.4, Agg_4 802.15.4 and Agg_6 802.15.4. Similarly, a significant increase is noticed for the other DC options with aggregated-802.15.4. The reason of this improved throughput is the better channel utilization through aggregation mechanism. We deal with periodic data of patient monitoring systems, where nodes generate many small packets in a short time. In 802.15.4, due to channel access overheads, packets start dropping from the queue, which affects the throughput. The other major reason of low throughput for this scenario is inefficient channel utilization, where a node gets a channel to transmit only a small packet and then again it need to contend. The aggregation mechanism provides a node opportunity to send multiple MPDU in a single channel access. 

**Figure 11:**
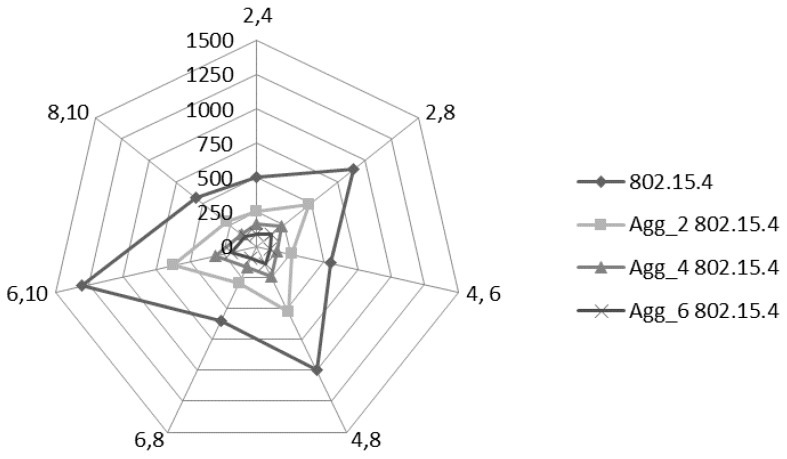
Average delay comparison.

Figure 11 provides the delay comparison of the similar scenario provided in Figure 10. All the DC options for non-aggregated 802.15.4 provide the average delay values up to 500 ms or greater. For the patient monitoring system the end-to-end delay must be under 250ms so that meaningful information is generated [2,4,8,30,33]. It is observed that the average end-to-end delay reduces with the increase in the aggregation levels. Although the Agg_2 802.15.4 level gives the delay values more than 250 ms for a few DC options i.e., 2/8, 4/8 and 6/10, but the Agg_4 802.15.4 and Agg_6 802.15.4 aggregation levels provide average delay within 250 ms for all the DC options. The main reason for the reduced delay is that the multiple packets can be received collectively instead of a single packet which reduces the overheads of the channel access mechanism and the header transmission. The channel access overheads include random backoff delay, two times CCA, SIFS and acknowledgement transmission time. Therefore, if a node sends six packets in single channel access instead one packet in one channel access, then it significantly reduces the overheads for the five packets. 

**Figure 12:**
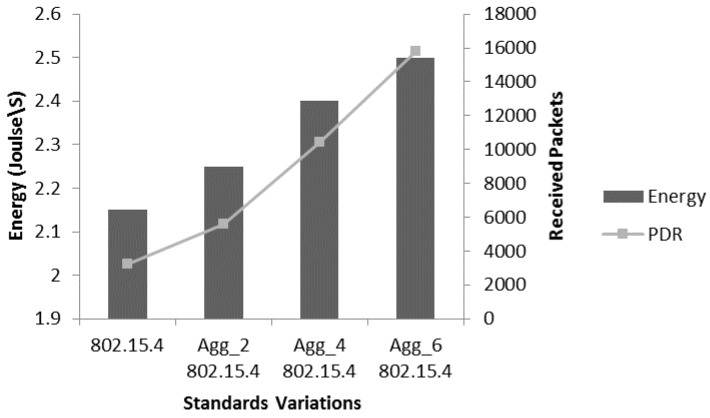
Energy consumption analysis of aggregation with received packets.

The efficient energy consumption for the WBASNs is considered as integral performance aspect. IEEE 802.15.4 provides a complete synchronization mechanism among coordinator and the network nodes [4,21]. The nodes know their awaken timing where they could content for the channel or simply send data in reserved GTS slots. DC mechanism is incorporated to adjust sleep and awaken timings. Figure 12 shows energy consumption analysis of different aggregation levels by considering number of received packets. For this analysis, we used the DC combination 2/4 (25%). A slight continuous increase in energy consumption is observed with the increase in aggregation levels. The reason for this increase is the increased packet size which takes more transmission time as the radio remains on. This marginal increase in the energy consumption is affordable if the significant increase is in the received packets which varies from 53,020 with Agg_4 802.15.4 to 14701 with Agg_6 802.15.4 aggregation. We already discussed the reasons for increased throughput. 

**Figure 13:**
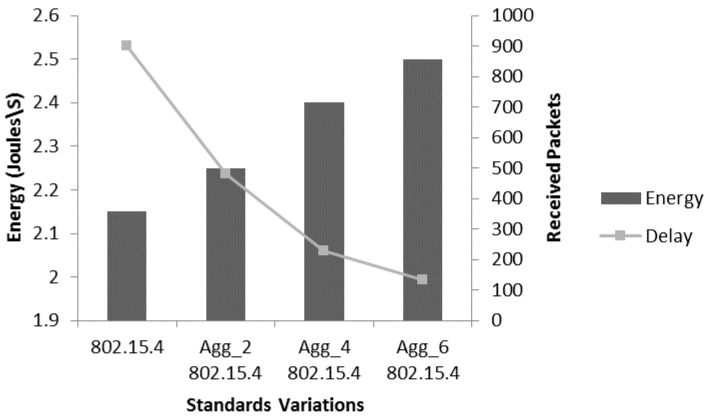
Energy consumption analysis of aggregation with delay.

Figure 13 describes energy consumption analysis of aggregation levels by considering the average end-to-end delay. The scenario and parameters are the same as mentioned in Figure 12. It is observed that the energy consumption increases slightly by increasing the aggregation level but on the same time the average end-to-end delay significantly reduces and come under 250 ms which is recommended for bio-medical sensor devices in patient monitoring systems. As the aggregation mechanism reduces the waiting time and transmission overheads, the average delay significantly reduces. Moreover, the use of block acknowledgement also contributes in reducing delay. Only one acknowledgement is sent for an A-MPDU which consists of maximum six MPDUs. In the case of high BER, there is probability of packet loss and then retransmission of the whole A-MPDU. However, the use of block acknowledgement make a receiving node capable to only sends a collective acknowledgement against those MPDUs which are received correctly. The sending node only retransmits the lost MPDUs which plays it role to reduce the average delay values.

**Figure 14:**
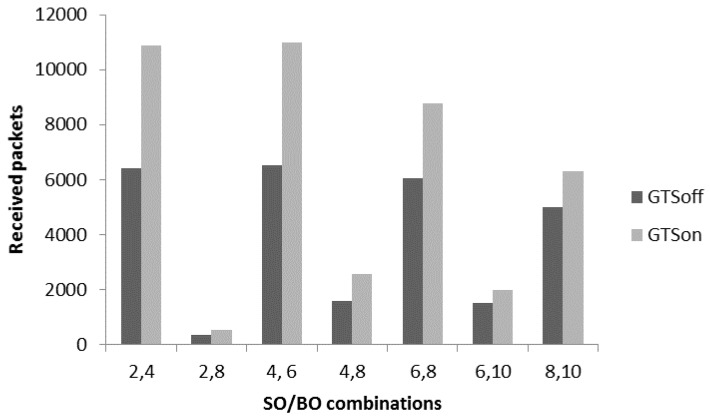
GTSon vs. GTSoff comparison.

It is noted that performance optimization is achieved in terms of received packets and average delay by using different levels of frame aggregation for slotted CSMA/CA in CAP mode. In the proposed patient monitoring system, we have two type traffic patterns: the first one is the high-periodic traffic where a huge number of small packets generate and other is low-periodic traffic which generates data with medium or low rate. To fulfil the requirements, we check the performance of the hybrid mode which means the nodes with high-periodic traffic characteristic are assigned the GTS slots, whereas the low-periodic traffic nodes use CAP. For the proposed patient monitoring system, ECG and EEG nodes are requested for three GTS slot for each. A coordinator can assign maximum seven GTS slots out of total 16 superframe slots. Figure 14 provides a comparative analysis of received packets between GTSon (where a few nodes request GTS slots) and GTSoff (all nodes use CAP). The different DC options are used to evaluate the performance of these modes. From Figure 14, a significant improvement in GTSon mode is observed for the number of received packets from 6000 to 10,500 for the DC option 2, 4. The reason is that the high-periodic traffic nodes get a guaranteed time slots in every superframe duration, so there are less chances of data loss due to contention and collision. Figure 14 shows that the hybrid scheme involving CFP and CAP is more suitable for the patient monitoring system with high-periodic and low-periodic traffic patterns.

**Figure 15:**
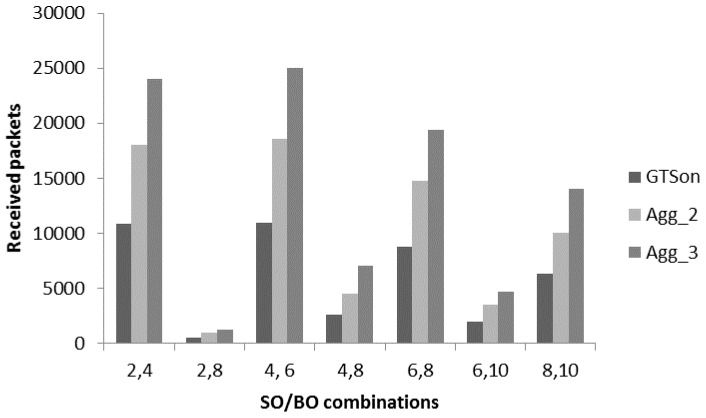
Received packets analysis for aggregation in GTSon mode.

Figure 14 proves that a hybrid (combination of CFP and CAP mode) sort of MAC mechanism fulfils the requirement of the proposed patient monitoring system. Therefore, to further improve the performance in terms of received packets we apply the proposed aggregation mechanism to check the full capacity of aggregation mechanism with GTSon mode. Figure 15 presents a comparative analysis of the received packets with non-aggregated GTSon mode and for aggregate-mode with different levels. A significant improvement can be seen with the frame aggregation levels. The frame aggregation mechanism significantly increases the number of received packets by reducing the delay. For example, with DC option 2, 4 if we use the 3rd level aggregation, then the maximum capacity goes from 10,000 to 24,000. It is concluded from the results of Figure 15 that for patient monitoring systems which use multiple sensors having different requirement works well with the hybrid channel access mode (GTSon). Moreover, with the help of frame aggregation mechanism, their performance can be further optimized to fulfil the requirements of patient monitoring systems which require time bounded services, reliable communication and efficient energy consumption.

**Figure 16:**
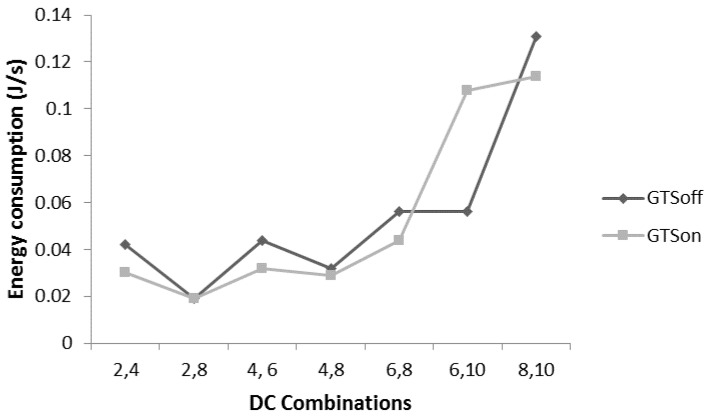
Maximum throughput analysis.

Figure 16 presents the energy consumption analysis for the scenario mentioned in Figure 15. Overall, the GTSon mode provides slightly better energy consumption with the bounded delay. The reason is that the nodes contend for fewer slots in the GTSon mode as compared to the GTSoff mode.

**Figure 17:**
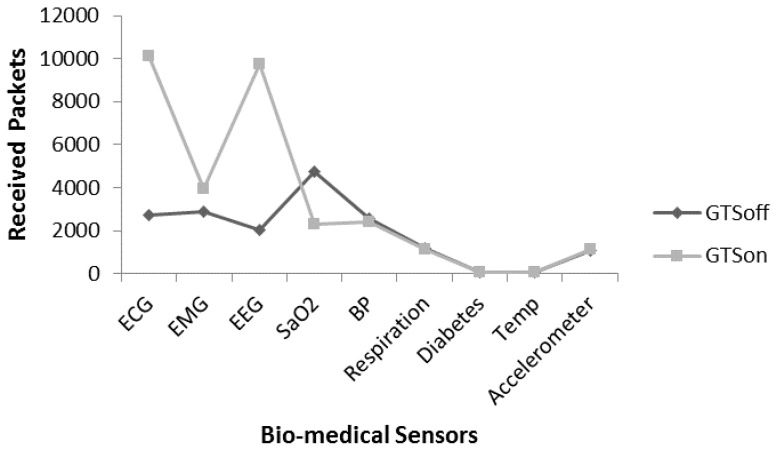
Received packet analysis for individual node without DC.

**Figure 18:**
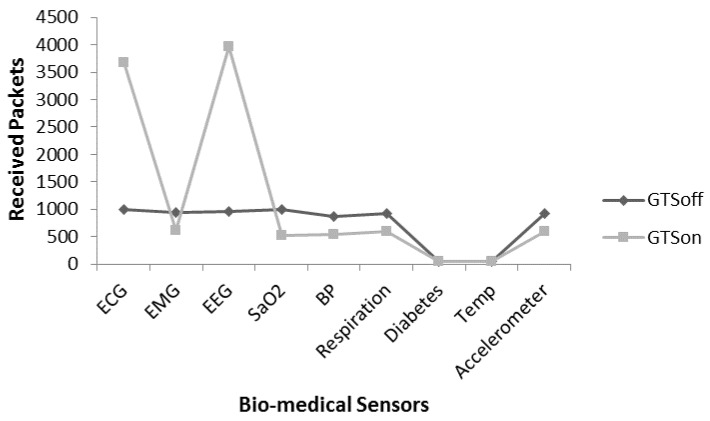
Received packet analysis for individual node with DC.

**Figure 19:**
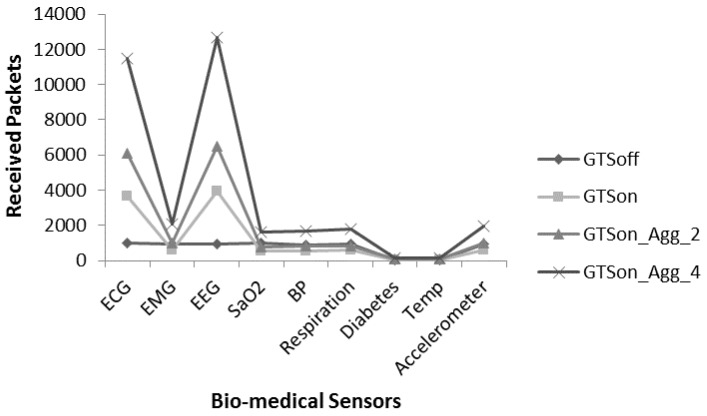
Received packet analysis for individual with aggregation and DC.

Figure 17, Figure 18 and Figure 19 provide an analysis of received packets for an individual node, that the number of packets received by the coordinator from an individual node. Figure 17 provides a comparative analysis of received packets with GTSon and GTSoff mode. In GTSon mode we assigned 3 GTS slots to ECG sensor and 3 GTS slot to EEG, the remaining seven nodes use slotted CSMA/CA in CAP mode. Figure 17 provides comparative analysis when nodes are 100% active, means with DC value 100%. It is noticed that ECG and EEG nodes provide high packet reception with almost 90% packet delivery ratio in GTSon mode, whereas their performance is low in GTSoff mode. The reason is that these nodes generate many small packets in a limited time and there is no guarantee whether they get a channel access or not as the other nodes are also contending; therefore, the packet lost ratio is high on queues due waiting time and limited space in queues. On the other hand when GTS mode is used for ECG and EEG they provide high number of received packets as in every superframe they get three GTS slots each. It is also noticed that in the hybrid mode (with usage of GTS slots) the node which does not use GTS can provide a lower number of received packets in comparison with a node which only uses slotted CSMA/CA like SpO2 sensor node. The reason for low received packets in the hybrid mode is that superframe is divided in 16 slots, GTS uses six slots (three for ECG and three for EEG) and 10 slots left for seven remaining nodes, these nodes content for the channel and SpO2 gets less number of channel accesses. On the other hand, in non-hybrid mode (all nodes use slotted CSMA/CA for channel access), nine nodes content for all the available slots, where SpO2 gets more slots than in the hybrid mode. The other remaining sensors including BP, respiration, diabetes, temp and accelerometer are sending data at low rate, therefore there is no critical issue involve with them. 

Figure 18 provides the analysis regarding suitability of a hybrid MAC for the proposed patient monitoring system. However, the DC mechanism is not considered in scenario relevant to Figure 17. Figure 18 provides the similar scenario in Figure 17 but with DC value 2/4 (25% active) to evaluate the performance by considering an energy efficient mechanism. It is noted that due to the usage of DC mechanism in hybrid mode the number of received packets reduces almost up to 50%. The reason is the less awaken interval of the nodes. The overall received packet rate for each senor is affected due to DC mechanism. 

To overcome the issue presented in Figure 18, we apply the frame aggregation mechanism. Figure 19 provides comparative analysis of received packets of individual sensors under four modes i.e., GTSoff, GTSon, GTSon_Agg_2 and GTSon_Agg_4. Moreover, the DC mechanism is considered with the value of 25%. It is noted that the frame aggregation mechanism increases the number of received packets significantly for each node.

We use IEEE 802.15.4 with operating band of 2.4 GHz that may also receive signals as interference from coexisting IEEE 802.11 deployed wireless networks and some unknown wireless networks. The device like Series 2 XBee-PRO S2B of Digi International which uses Ember (Silicon Labs, Global Headquarters, Digi International, 11001 Bren Road East, Minnetonka, MN, USA) EM250 as the radio chip which is IEEE 802.15.4-complaint transceiver and operates at 2.4 GHz ISM band with 250 kbps data rate. It provides 16 different operating channels. 

**Figure 20:**
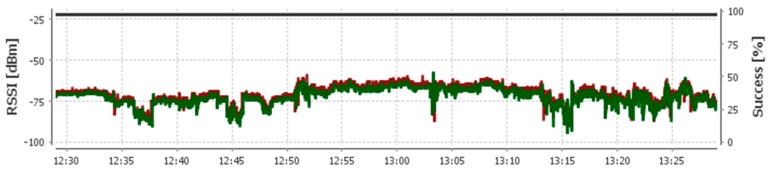
RSSI with interference.

The proposed aggregation mechanism provides efficiency in terms of delay, throughput and energy utilization to accommodate patient monitoring applications. In the above mentioned scenarios and analysis the data is collected from multiple sensors, for this scenario we consider that each patient consists of a WBASN and there are multiple WBASNs in a location like a hospital ward. For such scenario, the nodes may face interference from nearby WBANs and already deployed wireless LANs (WLANs) which works on 2.4 GHz band. To overcome this issue, all the WBASNs are configured at different communication channel, which means to cover multi-hop scenario, and the coordinator nodes of WBASNs operate on two channels i.e., one is for its internal network and other is for external communications (coordinator to central server). However, still there is some unwanted interference is observed through the received signal strength indicator (RSSI) which is due to human movement in the location as shown in Figure 20.

It is observed that there is some interference in the form of variations in RSSI values on a single channel due to human movement; however, it does not significantly affect the packet reception ratio (success [%]).

**Figure 21:**
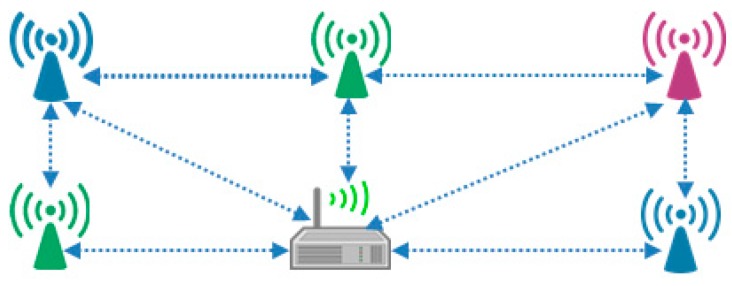
Multiple WBASN communication scenario.

**Figure 22:**
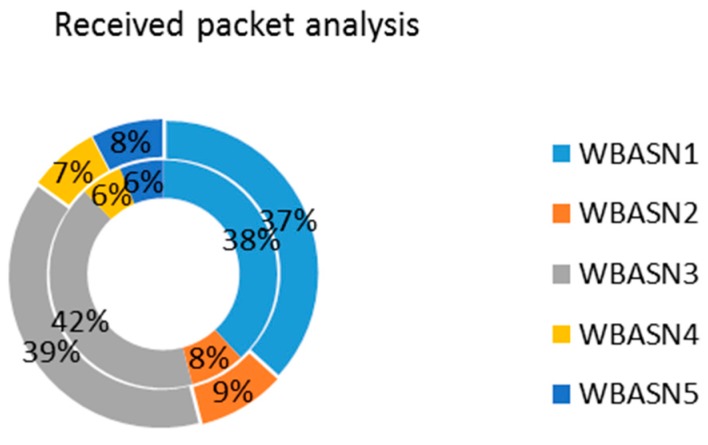
Received packet analysis.

The coordinator nodes can communicate to each other as well as with the central node. Moreover, the coordinator node can be used as a data forwarding node towards the central server. Figure 21 shows the scenario where coordinators of each WBASN communicate with the central server. Figure 22 shows received packet analysis at the central server/coordinator. The inner circle represents the aggregation mechanism at Agg_4, whereas the outer circle represents Agg_2. WBASN1 and WBASN3 have more data to send. Due to aggregation mechanism at the MAC layer, they get more percentage as explained earlier. WBASN3 and WBASN4 send 1/4th of data traffic as compared to WBASN1 and WBASN3. It is noticed that WBASN3 and WBASN4 packet reception is better than Agg_2 than Agg_4. The reason for this is their slow traffic generation rate.

## 5. Conclusions

Aiming at patient monitoring applications of WBASNs, a frame aggregation mechanism has been proposed at the MAC layer in this paper to improve the channel access mechanisms as well as to meet strict time deadlines, provide reliable data delivery and fulfill energy efficiency requirements at the same time. For the patient monitoring system, we have selected ten different bio-medical sensor devices, including a coordinator and deployed them in a star topology. The devices include ECG, EEG, EMG, accelerometer, gyroscope, pulse oximeter, blood pressure, temperature, barometer and heart rate monitoring, etc. The sensors periodically collect data from the body and send towards monitoring station through the coordinator node. The periodic, high prioritized and emergency data demand a different set of QoS when they are integrated in a patient monitoring system. To address the mentioned challenges, initially we have provided traffic pattern analysis of these devices to understand their communication requirements. From the traffic pattern analysis, it is observed that these biomedical sensor devices generate many small packets in a limited time. The IEEE 802.15.4 standard supports the maximum frame size up to 127 bytes including 25 bytes of MAC header and 102 bytes of payload and a successful packet transmission involves the overheads like random backoff delay, two times CCA, 802.15.4 header with each frame, SIFS and acknowledgement transmission time. The maximum packet size from these bio-medical sensor devices used in the patient monitoring system is 17 bytes for a single frame including headers. Each frame contents the channel to send the data, so a node which wins the channel transmits a small packet and for the next packet transmission, it needs to content the channel again. Therefore, on getting a channel, a node is under utilizing the available maximum packet size capacity which is 127 bytes for a MAC frame and only transmits up to 17 bytes. To address these issues, we have used the frame aggregation mechanism, which will send multiple MAC frames under a single PHY header within a single successful channel access. The concept of frame aggregation makes a huge difference in throughput and delay performance by efficiently utilization in channel access mechanism. The proposed mechanism is initially evaluated through numerical modeling. In the next step, simulation is conducted in NS 2.29 and CASTALIA 3.2 with OMNeT++. The evaluation is conducted for both modes i.e., CAP and CFP. The evaluating parameters include number of received packet, average delay and energy consumption. Various constraints are considered in the simulations including SO/BO combinations (DC), GTSon, GTSoff, network performance evaluation and individual node performance evaluation. 

## Figures and Tables

**Table 1:** Physiological signals: sampling rate, resolution, type and location [30].

Physiological Parameter	Sampling Rate (Hz) (Min–Max)	Sampling Resolution (Min–Max)	Type of Sensing Device	Location
ECG (per channel)	(100–1000)	(12–24)	Electrodes	Chest
EMG	(125–1000)	(12–24)	Electrodes	Muscles
EEG	(125–1000)	(12–24)	Electrodes	Head
Pulse oximeter	(100–1000)	(12–16)	Photodiode	Ear or finger
Blood pressure	(100–1000)	(12–24)	Pressure cuff	Arm or finger
Respiration	(25–100)	(8–16)	Elastic chest belt or Electrodes	Chest
Blood glucose	<0.01	(8–16)	Chemical	Skin
Skin temperature	<1 in 60 s	(16–24)	Thermistor probe	Wrist/arm
Activity	(25–100)	(12–24)	Accelerometers	Chest

**Table 2:** Comparative analysis of the proposed protocols.

Protocol	Standard	Access Scheme	Theme	Focused QoS
DQBAN [12]	IEEE 802.15.4	CSMA/CA	To enhance IEEE 802.15.4, two queues are introduced for successful channel access and data transmissions (collision resolution queue and data transmission queue).	R, C
D2MAC [11]	IEEE 802.15.4	CSMA/CA	Based on adaptive backoff time by using fuzzy logic.	D
U-MAC [8]	IEEE 802.15.4	TDMA	Urgency MAC provides priority for critical data for patient monitoring. U-MAC do not use option of retransmissions.	D
HUA-MAC [10]	IEEE 802.15.4	TDMA	Hybrid unified MAC only uses contention free period (CFP) for important data transmission, the normal traffic uses CAP.	D, R
Channel-MAC [9]	IEEE 802.15.4	TDMA	A single radio multi-channel TDMA MAC protocol to provide high reliability.	R
EELDC [21]	IEEE 802.15.4	TDMA	Energy efficient low duty cycle protocol provides low duty cycle values to increase the lifetime of the nodes.	E
CA-MAC [20]	IEEE 802.15.4	TDMA	Context aware MAC make nodes enable about energy of there and other nodes and help to increase the lifetime of the network.	E
BDD [24]	IEEE 802.15.4	TDMA	Battery dynamics driven protocol considers the current power conditions of the battery and useful for better energy consumption.	E
T-MAC [25]	IEEE 802.15.4	Hybrid	T-MAC allows the nodes to turn on the radios on pre-synchronized timings and turn off in case of no communication. T-MAC adjusts radio-on interval with the traffic rate, so there is no fix radio-on interval which makes T-MAC adaptive. It provides reliable and energy efficient communication.	R, E
S-MAC [14]	IEEE 802.15.4	Hybrid	S-MAC is considered as a predecessor of T-MAC and provides fixed radio-on intervals and solves idle listening problem. The coordinator assigns those wakeup intervals, after transmission the nodes go to sleeping mode. S-MAC gives low latency as due to synchronization there are less chances of collisions.	D, E
B-MAC [22]	IEEE 802.15.4	Hybrid	B-MAC [26] presents three bandwidth management schemes i.e., burst, periodic and adjusts bandwidth. B-MAC works in the environment where there is synchronization among sensor nodes and gateway nodes. Therefore, nodes only wakeup for transmit or receive the data. It uses Contention Free Period (CFP) to save energy. It also uses CSMA/CA in in Contention Access Period (CAP) for some specific scenario where network joining conditions are flexible.	E, T
X-MAC [15]	IEEE 802.15.4	Hybrid	X-MAC improves the B-MAC by making small preamble burst with destination address instead of long preamble.	E, D
PNP-MAC [26]	IEEE 802.15.4	Hybrid	Preemptive slot allocation and non-preemptive transmission MAC support medical applications through superframe adjustments.	D, E
VMAC [29]	IEEE 802.15.4	Hybrid	VMAC provides adaptive resource scheduling using asymmetrical architecture which provides reliability with bandwidth guarantee.	R, T
EMAC [27]	IEEE 802.15.4	Hybrid	EMAC integrates relay nodes to save the energy resources.	E
YNU-MAC [19]	IEEE 802.15.6	CSMA/CA	Uses SIFS, DIFS and backoff in contention window and provide efficient channel utilization with high data rates.	T, R
NICT MAC [10]	IEEE 802.15.6	TDMA	Suitable for star topology and is usable in beacon and beaconless mode and introduce the concept of group BAN superframe for scalability.	E, S
WiseMAC [34]	IEEE 802.15.6	TDMA	It is applicable for both for star and mesh topology and provides scalability.	S
IMEC [28]	IEEE 802.15.6	Hybrid	It uses enhanced slotted aloha by incorporating dual duty cycling and provides flexibility and power efficiency.	E
C-MAC [23]	IEEE 802.15.6	TDMA	C-MAC is made for mobile clusters for WBASNs and control the interference and collisions due to mobile nodes.	C
MEB- MAC [18]	IEEE 802.15.6	Hybrid	MEB-MAC protocol inserts a listening window in CFP and provides less delay to the medical applications.	D
MFS-MAC [16]	IEEE 802.15.6	Hybrid	Improved MAC protocol for WBASN to satisfy the energy consumption of implantable devices.	E
DT-SCS [35]	IEEE 802.15.4e	Hybrid	Performs decentralized time-synchronized channel swapping (DT-SCS) and reduces certain convergence and network utilization problems.	T, E
QL-MAC [36]	IEEE 802.15.4	Hybrid	A machine learing based approach that produces significant improvements in terms of network lifetime and throughput.	T,E

**Table 3:** Traffic pattern summary.

Physiological Parameter	Data Generation Interval	Data Generation (Bits)	Packet Size (Bytes)	Required Data Rate (Kbps)
ECG	4 ms	16	17	34
EMG	6 ms	11	16.3	19.6
EEG	4 ms	11	16.3	19.6
Pulse oximeter (SpO2)	10 ms	12	16.4	13.2
BP	10 ms	12	16.4	13.2
Respiration	40 ms	8	16	3.2
Blood glucose	250 s	0.032 (1 bit)	15.1	0.528
Skin temperature	60 s	0.266	16.98	2.27
Activity	10 ms	11	16.3	3.3

**Table 4:** MAC layer parameters and values [1,31].

Attribute	Value
$T_{\tau A}$	0.192 ms
$T_{\mathrm{SIFS}}$	0.192 ms
$T_{\mathrm{Ack}}$	0.864 ms
$T_{\mathrm{CCA}\;}$	0.128 ms
$T_{\mathrm{CW}}$	0.64 ms
$T_{\mathrm{symbol}}$	0.32 ms

**Table 5:** Simulation Parameters.

Parameters	Value
Number of nodes	Varies from 10–16
MAC	IEEE 802.15.4
Channel mode	Log Shadowing Wireless Model
Seed value	11
Frequency band	2.4 GHz
Data rate	250 kbps
Evaluation Criteria	Delay, Packet Delivery Ratio (PDR), Energy consumption
$T_{\mathrm{SIFS}}$	0.192 ms
$T_{\mathrm{Ack}}$	0.864 ms
$T_{\mathrm{CCA}\;}$	0.128 ms
$T_{\mathrm{CW}}$	0.64 ms
$T_{\mathrm{symbol}}$	0.32 ms
Simulation time	100–2000 s
